# Primary Biliary Cholangitis and Primary Sclerosing Cholangitis: Current Knowledge of Pathogenesis and Therapeutics

**DOI:** 10.3390/biomedicines10061288

**Published:** 2022-05-31

**Authors:** Ji-Won Park, Jung-Hee Kim, Sung-Eun Kim, Jang Han Jung, Myoung-Kuk Jang, Sang-Hoon Park, Myung-Seok Lee, Hyoung-Su Kim, Ki Tae Suk, Dong Joon Kim

**Affiliations:** 1 Department of Internal Medicine, College of Medicine, Hallym University, Chuncheon-si 24252, Korea; miunorijw@hallym.or.kr (J.-W.P.); jungheekim@hallym.or.kr (J.-H.K.); sekim@hallym.or.kr (S.-E.K.); con2000@hallym.or.kr (J.H.J.); mkjang@kdh.or.kr (M.-K.J.); sanghoon@hallym.or.kr (S.-H.P.); leemsmd@hallym.or.kr (M.-S.L.); hskim@kdh.or.kr (H.-S.K.); ktsuk@hallym.or.kr (K.T.S.); 2 Institute for Liver and Digestive Diseases, Hallym University, Chuncheon 200-010, Korea

**Keywords:** cholangiopathy, primary biliary cholangitis, primary sclerosing cholangitis

## Abstract

Cholangiopathies encompass various biliary diseases affecting the biliary epithelium, resulting in cholestasis, inflammation, fibrosis, and ultimately liver cirrhosis. Primary biliary cholangitis (PBC) and primary sclerosing cholangitis (PSC) are the most important progressive cholangiopathies in adults. Much research has broadened the scope of disease biology to genetic risk, epigenetic changes, dysregulated mucosal immunity, altered biliary epithelial cell function, and dysbiosis, all of which interact and arise in the context of ill-defined environmental triggers. An in-depth understanding of the molecular pathogenesis of these cholestatic diseases will help clinicians better prevent and treat diseases. In this review, we focus on the main underlying mechanisms of disease initiation and progression, and novel targeted therapeutics beyond currently approved treatments.

## 1. Introduction

Cholangiocytes are mature epithelial cells lining the biliary tree. Their functions include the secretion and modification of bile components and the transport of bile to the intestine. Dysfunction of cholangiocytes can lead to the development of various biliary diseases with a chronic–progressive course and often invalidating outcomes. This disease entity affecting the biliary epithelium is known as cholangiopathies. It is caused by diverse etiologies, including genetic, immune-mediated, infectious, drug-induced, idiopathic, malignant and vascular diseases [1]. The perturbed structure and function of cholangiocytes result in impaired bile formation and secretion. These syndromes comprising cholangiopathies have unresolved pathophysiological problems and significant unmet needs in clinical practice [2].

Primary biliary cholangitis (PBC) and primary sclerosing cholangitis (PSC) belong to the main subgroup of chronic cholestatic liver diseases. PBC is a progressive fibrosing cholangiopathy of the small intrahepatic bile ductules (diameter < 100 µm). The prevalence of PBC ranges from 1.91 to 40.2 per 100,000 inhabitants in different geographic areas [3]. In 2015, the nomenclature of PBC was changed from primary biliary cirrhosis to remove the “cirrhosis stigma” and more accurately reflect the disorder and its natural history [4,5]. Although a predisposing genetic background along with infective, immunological, and environmental triggers have been proposed to elucidate the onset of the disease, the etiology of PBC is still unclear [6,7,8].

PSC is also characterized by the progressive idiopathic stricturing of the biliary system, typically leading to end-stage liver diseases such as cirrhosis and colonic or hepatobiliary malignancy. The prevalence of PSC ranges from one to 16 per 100,000 worldwide [3,9], with an annual incidence of 0.5–0.75 cases per 100,000 [9,10]. The pathogenesis of PSC remains uncertain; however, similar to PBC, it appears to be multifactorial, with environmental triggers leading to cholangiocyte damage and an aberrant and exaggerated cholangiocyte immune response promoting clinical disease in immunogenetically susceptible persons.

Both PBC and PSC are considered immune-mediated cholangiopathies and have features of portal inflammation, biliary tract injury, and sequential fibrosis and cirrhosis development, leading to end-stage liver failure. Although these disorders present common clinical features, PSC is characterized by damage of medium to large extrahepatic and intrahepatic bile ducts, whereas PBC chiefly targets small intrahepatic bile ducts [11,12]. They are uncommon or even rare; however, these diseases often cause considerable morbidity and mortality. The largely unknown etiology and disease mechanisms of PBC and PSC make curative therapies unavailable.

Thus, in this review, we address the mechanism of functional loss of cholangiocytes in the initiation and progression of PBC and PSC, together with the treatments based on currently ongoing studies.

## 2. Cholangiocyte Pathobiology

Cholangiocytes line a complex network of interconnecting tubes extending from the canals of Hering (CoH) in the liver to the duodenum. CoH begins in the lobules, consists partially of hepatocytes and cholangiocytes, and carries bile from the bile canaliculi to terminal bile ducts in portal tracts [13]. Even though cholangiocytes compose a minor part (3–5% of all liver cells) of the liver, they are essential in bile generation, a life-sustaining function of the liver [14]. The fact that cholangiocytes account for 3–5% of the total liver cell population and generate 25–40% of the total bile flow in humans, which indicates how active these cells are when transporting water and HCO_3_^−^ into the bile duct [15,16,17].

Cholangiocytes represent heterogeneity with regard to morphological, biochemical, and functional aspects [18]. Immature cholangiocytes within the CoH, and from the intrahepatic and extrahepatic peribiliary glands, are poorly differentiated, and are considered progenitor cells that participate in epithelium restoration and tissue regeneration; however, cholangiocytes gradually obtain a greater degree of differentiation along the biliary tree (from small to large bile ducts) regarding cell polarity, expression of receptors and transporters, and response to hormones [18,19]. Cholangiocytes are classified into small and large cholangiocytes, and each consists of small and large intrahepatic bile ducts, respectively. Large cholangiocytes take part in the alteration of bile composition and volume through secretory and absorptive processes which thoroughly regulated by molecules such as hormones, peptides, and neurotransmitters [20]. In contrast, small cholangiocytes can change their phenotype in response to exogenous or endogenous -stimuli, including microorganisms, toxic material, drugs, and hormones, which consequently participate in the inflammatory response during biliary tract injury [20,21], and function as liver progenitor cells in certain circumstances [22].

Bile, consisting mainly of bile salt, phospholipids, cholesterol, conjugated bilirubin, electrolytes, and water, is a physiological aqueous solution and secretory fluid produced by the hepatobiliary system. Bile contains various components which contribute to health by aiding digestion, maintaining enterohepatic circulation, and supporting the elimination of harmful molecules from the body. Within the ductal lumen, bile is modified via activities at the apical plasma membrane domain of cholangiocytes. Additionally, via tight junctions and immunoglobulin, A (IgA) secretion, and barrier formation, cholangiocytes protect themselves from potentially damaging molecules and microorganisms in bile, and can access the immune and vascular systems through the basolateral plasma membrane domain. These complicated processes are regulated by extracellular signals, biliary constituents (bile acids, glucose, vesicles), and physical forces such as flow and pressure [15].

Cholangiocytes can be affected during liver injury and participate in various liver diseases’ pathobiology. Additionally, cholangiocytes can also have a role in liver regeneration when hepatocyte regeneration is impaired [23]. Detailed molecular mechanisms responsible for cholangiocyte dysfunction in PBC and PSC will be reviewed in the following sections.

## 3. Primary Biliary Cholangitis

### 3.1. Pathogenesis of PBC

#### 3.1.1. Genetic Factor

PBC can be triggered by environmental factors such as infectious diseases and harmful chemicals in genetically susceptible individuals [6]; therefore, genetic factors are thought to play a substantial role in disease onset. There was a higher concordance rate in monozygotic twins than in dizygotic twins, and familial clustering of patients with PBC (relative risk of 9.13–10.5 in first-degree relatives compared with 1.66 in fifth-degree relatives), which suggests that genetic factors contribute to the occurrence of PBC [24,25,26]. Although the genetic correlations are lower than those seen in other autoimmune diseases, large-scale genome-wide association studies have recently indicated multiple genes affecting the susceptibility to PBC in human leukocyte antigen (HLA) and non-HLA loci. The HLA complex at chromosomal position 6p21 includes the most polymorphic genes in the human genome [27]. The products of the classical HLA class I (A, B, and C) and class II (DR, DQ, and DP) genes contain cell surface glycoproteins participating in the binding and presentation of self or non-self peptides to T-cell receptors. Class I molecules are recognized by CD8+ cytotoxic T cells presenting as endogenous peptides, whereas class II molecules are recognized by CD4+ helper T cells presenting as processed peptides from exogenous pathogens [28]. The extent to which endogenous and exogenous peptides bind to HLA molecules is determined by allelic polymorphisms.

HLA class II alleles have been associated with PBC onset for decades. Previous studies have shown that PBC is related to HLA DR*08 as a predisposing allele and HLA DRB1*11 and *13 alleles as protective alleles [29,30]. Although HLA alleles are crucial in determining the susceptibility of PBC via alteration of autoantigen presentation, HLA alone does not clarify the whole genetic predisposition to PBC. In fact, 80–90% of patients with PBC do not represent the most common HLA susceptibility alleles [28]. Genome-wide association study (GWAS) findings denoted risk loci including *interleukin* (*IL*)*12A*, *IL12RB*, *interferon regulatory Factor 5* (*IRF5*), *transportin 3* (*TNPO3*), *transcription factor Spi-B* (*SPIB*), *tumor necrosis factor superfamily*, *member 15* (*TNFSF15*), and *POU domain class 2-associating Factor 1* (*POU2AF1*) [31,32,33]. Through interaction with death receptor 3 (DR3), TNFSF15 promotes T-cell expansion and induces apoptosis in cells that overexpress DR3. Additionally, synergistic interaction with IL-12 and IL-18 ultimately promotes interferon-gamma production. The reason why much of the heritability of PBC remains uncertain may be explained by the “missing heritability”, such as DNA methylation, histone modification, and noncoding RNAs (i.e., miRNA and lncRNA) that cannot be captured by GWAS. For example, polymorphisms of solute carrier family 4 member 2(SLC4A2)/anion exchanger 2 (AE2) genes were associated with disease progression in a case-control study [34] but not in GWAS. In addition, telomere dysregulation in biliary epithelial cells (BECs) may be involved in disease onset. A deficiency in sex chromosomes and skewed gene expression in the X chromosome may explain the female predominance in PBC [35]. A previous study demonstrated that through X-chromosome profiling data of PBC patients, there are distinct DNA methylation patterns in CD4, CD8, and CD14 cells; moreover, demethylation of the C-X-C chemokine receptor 3 (CXCR3) promoter leads to elevated expression of CXCR3 in CD4 T cells in early-stage PBC patients [36]. CXCR3, which is expressed mainly on activated T lymphocytes, natural killer (NK) cells, and some epithelial cells and endothelial cells, plays a key role in inducing leukocyte trafficking, cytoskeletal changes, and chemotactic migration. To investigate the genetic variation underlying the progression of the disease and therapeutic response, further studies are necessary.

#### 3.1.2. Anion Exchanger Deficiency in PBC

Similar to other epithelial cells, cholangiocytes are polarized cells that enable the secretion of bicarbonate (HCO_3_^−^) and water to the ductular lumen. Thus, fluidizing and alkalizing canalicular bile can be formed [15,16]. The bile epithelium is protected from bile salts through the secretion of HCO_3_; therefore, a damaged “biliary bicarbonate umbrella” is considered a common pathology of fibrotic cholangiopathy [37]. Cholangiocytes react to pathogen associated molecular patterns (PAMPS) and damage associated molecular patterns (DAMPS) through the pattern recognition receptors (PRRs). This reaction leads to stimulation of the NF-κB-dependent cytokines/chemokines secretion. Pro-inflammatory cytokines and chemokines inhibit biliary fluid and bicarbonate secretion by interfering with cAMP-dependent ion transport mechanisms of cholangiocytes [38]. AE2, an electroneutral chloride (Cl^−^)/HCO_3_^−^ exchanger, has been reported to play a central role in maintaining a “biliary bicarbonate umbrella” for protecting biliary mucosa. Bile salt is negatively charged on the outer leaflet of the plasma membrane, where it can be protonated by attracting protons. Consequently, the protonated bile salts are not polar and enter cells freely through diffusion, resulting in apoptosis. The main function of the biliary bicarbonate umbrella is that of the alkaline barrier, which is maintained by biliary HCO_3_ secretion, and sets bile salts in their polar, deprotonated, and membrane–impermeable state; therefore, dysfunctional AE2 may be related to the pathogenesis of PBC [39,40,41]. In addition to several studies indicating that AE2 was reduced in PBC, a recent study reported that downregulation of AE2 may sensitize BECs to apoptotic insults activating soluble adenylyl cyclase (sAC) [39]. sAC, an evolutionarily conserved bicarbonate sensor, plays a key role in regulating bile salt-induced apoptosis [42]. Reduced AE2 expression leads to a decrease in the bicarbonate secretion and bicarbonate accumulation in the cells. Increased intracellular bicarbonate modulates bile salt induced apoptosis by increasing sAC activity. Decreased bicarbonate secretion allows more bile salts to enter cells, releasing intracellular Ca^2+^ stores, thereby increasing sAC activity. In vitro studies reported that inhibition of sAC reversed sensitization to bile salt-induced apoptosis and prevented bile salt-induced apoptosis altogether [39,40]. Motoko et al., have previously reported that uncontrolled autophagy may play an important role in the pathogenesis of PBC by causing autoimmune processes through aberrant expression of mitochondrial antigens such as the pyruvate dehydrogenase complex, E2 (PDC-E2) and promoting cellular senescence in BECs in the biliary tract in PBC [41,43,44]. More recently, it was disclosed that the decreased expression of AE2 was closely correlated with the abnormal expression of PDC-E2 and autophagy-related markers LC3 and p62 [45]. Furthermore, AE2 knockdown was reported to induce cellular senescence [45,46]. Interestingly, dysregulated autophagy can induce cellular senescence [47]; thus, it is likely that cellular senescence can be induced through unregulated autophagy due to AE2 downregulation in PBC. Senescent BECs express diverse chemokines and cytokines with a senescence-associated secretory phenotypes in PBC and PSC, which are involved in inflammatory cell infiltration and fibrosis in cholangiopathies [43,48,49,50].

In addition, it is interesting that allelic variations in the AE2 gene have a meaningful association with the disease progression rate in PBC under ursodeoxycholic acid (UDCA) treatment [34].

#### 3.1.3. Anti-Mitochondrial Antibodies (AMA) in PBC

The presence of disease-specific autoantibodies, that is, serum AMA, suggests that autoimmunity is one of the central mechanisms of PBC [51]. AMA was first detected for a non-organ-specific ATPase-associated antigen called M2, and it was primarily directed to PDC-E2 [52]. AMA is considered as the typical characteristic of PBC and is present in 90–95% of patients [53]; however, it can be found in less than 1% of healthy subjects. AMA targets lipoic acid-containing immunodominant epitopes, especially the E2 subunits of the 2-oxoacid dehydrogenase complex enzymes, including PDC-E2. For unknown reasons, PDC-E2 is abnormally expressed in the luminal surface of the bile duct epithelial cells of PBC patients, resulting in a pathogenetic process, so-called “autoimmune epithelitis” [54]. CD8+ T cells recognize this epitope, resulting in subsequent bile duct injury and accumulating bile acids to toxic concentrations [55,56]. The T-lymphocyte-mediated destruction of small bile ducts is followed by secondary damage of hepatocytes from the accumulation of potentially toxic molecules such as bile acids, which are normally secreted into the bile. Naturally, generated bile acids (cholic acid, chenodeoxycholic acid, and deoxycholic acid) are all detergents and can lyse cell membranes if present at a substantially high concentration, which is often reached in cholestasis [56].

Although AMA has diagnostic value, it has no prognostic value. In other words, AMA titer and subtypes are not associated with disease severity and outcome [57,58,59]. Although there was a report that treatment with UDCA may decrease the AMA titer, it is controversial whether the AMA titer is associated with treatment response [60,61].

AMA-negative PBC accounts for 5–10% of PBC cases [54,62]. PBC-specific anti-nuclear antibodies (ANA) (anti-GP210 and/or anti-SP100) might help to diagnose AMA-negative subjects. Interestingly, anti-SP100 and anti-GP210 are specific for PBC and correlate with disease severity [63].

#### 3.1.4. Immune Response in PBC

The immune system plays a central role in PBC pathogenesis, and various immune cells have been shown to infiltrate the portal tract areas of PBC patients. The primary cause of PBC relates to the loss of immune tolerance to PDC-E2 [64]. PDC-E2 is located on the inner mitochondrial membrane and contains a lipoic acid–lysine bond, which is essential for antigen recognition and immune cell activation [65,66]. Disease-specific AMAs target immunodominant epitopes, chiefly PDC-E2. Aberrant modification of mitochondrial PDC-E2 occurs within apoptotic BECs, and characteristic apoptotic blebs containing immunologically intact PDC-E2 are released. The immunogenic complex is recognized by circulating AMAs; as a result, antigen–antibody complexes are formed [67]. Then, autoantigen–AMA complexes are recognized by innate immune cells such as macrophages, and the disrupted immunotolerance of the liver leads to the further recruitment of various immune cells into the liver [67,68].

BECs can also directly act as antigen-presenting cells to present CD1d-restricted antigens to invariant natural killer T (iNKT) cells, resulting in the activation of this key immune cell subset [69]. In addition to enhancing immune activation, BEC can sustain an inflammatory profile in PBC, which is achieved by secreting several chemokines and recruiting the corresponding immune cells in the liver [48,70]. Once the immune response is set up against aberrant autoantigens expressed on cholangiocytes, PDC-E2-specific autoreactive CD4 T and CD8 T cells are selectively abundant in the livers of PBC patients [71]. Additionally, autoreactive B cells may act as important antigen-presenting cells via the uptake and presentation of autoantigens to T cells [72], which ultimately activate autoreactive B cells to release more AMA [73], thus developing a positive feedback loop that induces BEC injury in PBC.

During PBC progression, a shift from the Th1 to Th17 response occurs. Th17 cell activation increases with disease progression.; thus, maximal Th17 activation can be a feature of the progressive disease stage [74,75]. In addition to promoting inflammation, Th17 cells are known to perform profibrotic functions such as interleukin 17A (IL-17A), as a mechanism to promote the proliferation of hepatic stellate cells; however, this pathologic mechanism has not been proven in PBC [76]. An in vitro study demonstrated that mucosal-associated invariant T (MAIT) cells, another main source of IL-17 in later stages of PBC, induced a profibrotic and activated phenotype of human hepatic stellate cells (HSCs) [77,78,79]. Subsequently, immune-mediated mechanisms involving autoreactive T cells contribute to the development of chronic liver inflammation and direct bile duct injury by secreting a wide variety of pathologic factors.

#### 3.1.5. Gut Microbial Profile in PBC

The human gut harbors a complicated ecosystem of trillions of microbial cells, called the gut microbiome, contributing to essential functions such as regulating metabolism and immunity [80,81]. Accumulating evidence implies potential links between the gut microbiome and PBC [82,83,84]. PBC can change the gut microbiome by causing intestinal motility disorders, immunologic derangement, bile secretory defects, and portal hypertension [84,85]. The interactive relationship between the gut and the liver, called the “gut-liver axis”, is established by the portal vein, which enables the transport of gut-derived products directly to the liver [86].

The biliary epithelium expresses toll-like receptors (TLRs). Once various ligands, including microbial products such as lipopolysaccharide (LPS), bind to TLRs, cellular injury occurs through the proinflammatory nuclear factor-κB pathway and IL-8 and CX3C-chemokine ligand 1 (CX3CL1) release, which facilitates the recruitment of effector lymphoid cells into portal tracts in the livers of PBC patients [87,88]. In contrast, MAIT cells, a novel subset of innate-like T cells, have a critical role in protecting the biliary tree from microbial triggers; thus, MAIT cells are referred to as a “biliary firewall” [89]. Under healthy conditions, MAIT cells that are abundant in the portal vein are activated by antigen exposure and enhance the local immune response to suppress pathogens [77]; however, an amount of intrahepatic MAIT cells is decreased in PBC, therefore, the protective role of MAIT cells in the maintenance of biliary integrity is limited, especially after exposure to bacterial pathogens [90]. Interestingly, a recent study showed that even after response to UDCA treatment, the amount of MAIT cells did not normalize, which suggested the mechanism of the disease progresses in spite of the treatment response in PBC [79,89].

Additionally, portal plasma cells produce liver-derived secretory IgA, which is secreted into the lumen via bile. Secretory IgA is also able to contribute to protecting the biliary tree from microorganisms by agglutinating or entrapping bacteria, neutralizing bacterial toxins, altering the virulence of bacteria via disruption of gene expression and inhibiting bacterial access to enterocytes [91]. Compared with healthy individuals, PBC patients have reduced secreted IgA from duodenal enterocytes. Moreover, relevant localization of IgA to the basolateral membrane and expansion of tight junctions potentially serving as entrances to the bile tree were observed [92,93].

Distinct fecal microbial features in patients with PBC include increased colonization of *Enterobacteriaceae*, *Pseudomonas*, *Veillonella*, and *Clostridium*, and decreased *Oscillospira* and *Suterella* [94,95]. In a previous experimental study, long-term bacterial exposure in normal mice was associated with autoantibody production and histological signatures resembling PBC [96]. Recently, Tang et al., reported differences in the fecal microbiota from PBC patients naïve to UDCA compared with healthy controls [97]. In PBC patients, the gut microbiota richness was significantly reduced. At the phylum level, Bacteroidetes spp. were significantly decreased, whereas Fusobacteria and Proteobacteria spp. were overrepresented. At the genus level, Bacteroidetes spp., *Suterella*, *Oscillospira* and *Faecalibacterium* were significantly decreased in patients, whereas eight genera (*Haemophilus*, *Veillonella*, *Clostridium*, *Lactobacillus*, *Streptococcus*, *Pseudomonas*, *Klebsiella*, and an unknown genus in the family *Enterobacteriaceae*) were significantly increased [98]. Another study, including early-stage PBC patients and healthy controls, demonstrated that the guts of PBC patients were depleted of some potentially beneficial bacteria, such as Acidobacteria, *Lachnobacterium* spp., *Bacteroides eggerthii*, and *Ruminococcus bromii*, but were plentiful in some bacterial taxa including opportunistic pathogens, such as γ-Proteobacteria. [99]. As a result of that study, it was suggested that the alteration of the gut microbiome could be critical for the onset or development of PBC by interacting with metabolism and immunity. Interestingly, Tang et al. found that PBC-associated dysbiosis was partially reversed during UDCA treatment [97]. This finding suggests that modifications of specific bacterial species—for example, those sensitive to bile salt concentration and those involved in bile acid metabolism—may occur after UDCA treatment. Bile acids, which are important metabolites of the microbiome, can modify the composition of the gut microbiota directly or indirectly through activation of the innate immune system [100,101]. Considering the results of recent studies, once PBC occurs together with dysbiosis of the gut microbiota, a vicious cycle may be established, leading to harmful and even fatal outcomes in patients.

### 3.2. Currently Approved Disease-Modifying Therapies for PBC

#### 3.2.1. UDCA

Until recently, UDCA was an accepted first-line treatment for PBC. UDCA targets bile secretion and bile acid synthesis to improve biochemical markers, histological findings, complication rates, and transplant-free survival [102].

UDCA consists of a hydrophilic dihydroxy bile acid originating in the colon via bacterial 7β epimerization of chenodeoxycholic acid (CDCA) and is present in low concentrations in human bile acids. UDCA is passively absorbed through the colonic mucosa and then enters the circulating bile acid pool [103]. Although UDCA does not alter total bile acid amount, serum cholesterol levels, or bile phospholipid production, UDCA promotes the metabolic conversion of cholesterol to bile acids and reduces the cholesterol fraction of biliary lipids [104]. The suggested mechanisms of UDCA are as follows: (1) enrichment of the hydrophilic bile acid pool with exogenous UDCA that replaces endogenous hydrophobic toxic bile acids, which often occurs in cholestasis; (2) stimulation of bile secretion via upregulation of AE2 expression on the surface of BECs; and (3) immune modulation through a reduction in hepatocellular and biliary expression of major histocompatibility complex (MHC) class I and MHC class II proteins, possibly reducing adaptive immunity-mediated injury [105]. Additionally, reduced toxic bile salt disruption of cholesterol-rich membranes, especially of highly exposed cholangiocytes, leads to the stabilization of cell structures such as plasma membranes and mitochondria; thus, cytoprotective effects can be induced by UDCA [103]. Additionally, the subcellular anti-apoptotic pathway might be activated by UDCA, which possibly promotes mitochondrial membrane stability via the inhibition of the Bcl-2-associated X protein (BAX) translocation, a pro-apoptotic protein related to the initiation of core apoptotic pathways within mitochondria in hepatocyte cell lines [103]. UDCA might also suppress deregulated cholangiocyte autophagy pathways related to intense endoplasmic reticulum stress caused by toxic hydrophobic bile acids, as observed in cultured BECs and livers of PBC patients [43,44].

UDCA at a dose of 13–15 mg/kg/day has been recommended to decrease the progression of PBC [102,106]. To define the biochemical response, several criteria have been proposed (Table 1). Most studies have indicated that alkaline phosphatase (ALP) and total bilirubin are the two most meaningful variables in evaluating theUDCA response. Above this, prediction of the response before the commencement of UDCA is attempted in the UK and Italy [107]. Bilirubin, ALP, transaminase, age, and lag time from diagnosis to treatment are suggested as parameters predicting the response to UDCA. Up to 40% of patients with PBC will denote a suboptimal biochemical response to UDCA [108]; therefore, other treatment options are necessary.

#### 3.2.2. Obeticholic Acid (OCA)

There is no consensus on therapies for patients with suboptimal biochemical responses to UDCA. OCA is the first novel adjunctive licensed agent beyond the traditional use of UDCA alone in PBC. OCA, a semisynthetic hydrophobic bile acid analog, is highly selective for the farnesoid X receptor (FXR) because bile acid becomes more hydrophobic and a more potent FXR activator [116]. OCA with enhanced hydrophobicity through modification of CDCA showed 100-fold improved FXR potency compared with CDCA [117]. FXR is abundantly expressed in the liver and enterocytes. Understanding the mechanism of action of FXR agonism in chronic cholestasis has resulted in the development of OCA.

Primary bile acids such as cholic acid and CDCA bind FXR and downstream signaling pathways, ultimately inhibit the transcription of *CYP7A1* and the potent suppression of bile acid synthesis [4]. In reabsorption from the intestinal lumen, bile acids also activate FXR in ileal enterocytes. FXR activation in these cells results in the expression of fibroblast growth factor 19 (FGF19), which enters the portal circulation and binds to cell surface fibroblast growth factor receptor 4 (FGFR4) on hepatocytes, resulting in the suppression of *CYP7A1* expression and bile acid synthesis [118,119,120]. In addition, activation of FXR promotes the export of bile acids out of hepatocytes and enterocytes and hepatocyte and ileal reuptake [121,122].

A double-blind phase III clinical trial from the PBC OCA International Study of Efficacy (POISE) group demonstrated that 12 months of OCA therapy (add-on to UDCA or as monotherapy) led to a better biochemical response than the placebo group in nearly half of the PBC patients who were prior biochemical non-responders or intolerant to UDCA. A recent 3-year interim analysis showed significantly decreased ALP and bilirubin after 48 months of OCA treatment compared with the baseline [123,124].

### 3.3. Novel Therapies Currently under Investigation for PBC

#### 3.3.1. Peroxisome Proliferator-Activated Receptor (PPAR) Agonists

PPAR, a nuclear hormone receptor, participates in multiple metabolic processes, including the regulation of bile acid homeostasis [125]. PPAR agonists have been known to induce beta-oxidation gene expression, decreasing oxidative stress and inflammation in the liver while increasing the secretion of favorable adipokines [126]. PPAR agonists also inhibit hepatic bile-acid transportation [127]. PPAR has three distinct isoforms, α, δ and γ. PPARα, which is abundantly present in the liver, induces the expression of numerous genes related to lipid and bile-acid metabolism and the downregulation of genes in immune-related pathways [128]. In cholangiocytes, PPARδ agonism has been known to coordinate cholesterol flux and bile acid metabolism and has a role in apoptotic cell elimination via macrophages, reducing the autoimmune response against self-antigens released by dying cells [129,130]. PPARγ, found in the intrahepatic biliary epithelium, suppresses proinflammatory cytokine production and contributes to bile acid homeostasis [131]. PPARγ levels are reduced with bile duct injury. A previous study reported that administration of PPARγ ligands significantly reduced portal inflammation and the number of T cells in a mouse model of PBC [132]. Reports from the USA, Europe, and Asia demonstrated good efficacy of fenofibrate, an oral medication of the fibrate class (PPARα ligands), in PBC patients with a suboptimal response to UDCA [133,134]. Although the FDA approves fibrates only as lipid-lowering agents, their effect on lowering biochemical markers of cholestasis, such as ALP and γ-glutamyltransferase (GGT) levels, has been reported [135].

Compared with fenofibrate, which is specific to PPARα, bezafibrate is a pan (α, β/δ, γ) PPAR activator. Previous studies demonstrated that bezafibrate combined with UDCA could significantly decrease ALP, GGT, alanine aminotransferase (ALT), IgM, triglyceride, and total cholesterol levels [136,137]. In recent studies published after 2018, bezafibrate, combined with UDCA in PBC patients unresponsive to UDCA monotherapy, improved liver biochemistry and prognostic scores (e.g., UK-PBC and GLOBE scores) for long-term prognoses [94,138]; however, unfortunately, adverse reactions with bezafibrate-UDCA combination therapy are more frequent than UDCA monotherapy, including polydipsia, exacerbation of itching, arthritis, elevated serum creatinine levels and muscle pain, leg edema and gastrointestinal discomfort [136,139].

Recently, data on the efficacy and safety of elafibranor, a novel PPARα and PPARδ agonist, for treating noncirrhotic patients with PBC and an incomplete response to UDCA were published [140]. Patients receiving a dose of 80–120 mg of elafibranor showed a significant decrease in ALP compared with the placebo group. Additionally, adding the dual PPARα and PPARγ agonist, saroglitazar, to UDCA resulted in a significant decrease in ALP levels, showing an acceptable toxicity profile [141].

As a second-line treatment, seladelpar, a potent and selective PPAR δ agonist, has been investigated [142,143]. The phase 3 ENHANCE study was conducted in patients with PBC who did not respond to first-line treatment. This clinical trial was terminated early because of an unexpected histologic finding (nonalcoholic steatohepatitis); however, the finding was revealed to be unrelated to seladelpar. When a blind analysis following termination was performed, the treatment response rate was significantly higher in the seladelpar group. Additionally, seladelpar improved PBC patients’ pruritus and quality of life [144]; therefore, ongoing studies on the efficacy and safety in PBC patients are noteworthy.

#### 3.3.2. Budesonide

Budesonide, the second generation of corticosteroids, has a high first-pass metabolism within the liver; thus, fewer systemic adverse effects is reported than conventional glucocorticosteroids. Previous studies demonstrated that budesonide (6–9 mg/day) added to UDCA (15 mg/kg/day) in PBC patients showed better biochemical and histological improvement than UDCA monotherapy [145]; however, a recent small-scale randomized clinical trial demonstrated that budesonide (9 mg/day) added to UDCA (12–16 mg/kg/day) for 36 months failed to attain histological improvement in PBC patients with a suboptimal response to UDCA monotherapy [146]. Nevertheless, a combination of budesonide and UDCA might be an effective therapy for PBC patients; thus, further investigation is essential to evaluate the efficacy on long-term clinical outcomes, including mortality and the requirement for liver transplantation.

#### 3.3.3. Fibroblast Growth Factor (FGF) 19 Analog

FGF19, an endocrine hormone, is induced in the intestine with the activation of FXR [147]. In the liver, FGF19 suppresses the expression of CYP7A1, the gene encoding cholesterol 7α-hydroxylase, which catalyzes the first and rate-limiting step in the classic pathway of bile acid synthesis [148]. Administration of FGF19 has been reported to reduce liver damage in mouse models of intrahepatic and extrahepatic cholestasis [149,150]; however, the therapeutic potential of FGF19 is limited because of concerns about tumorigenicity, such as the development of hepatocellular carcinoma induced by ectopic overexpression of FGF19 in mice [151]. To overcome this defect, NGM282, a nontumorigenic engineered analog of FGF19, was developed to treat PBC, and MGM282 administration for 28 days showed a significant response in ALP and transaminase levels compared with placebos in PBC patients with an inadequate response to UDCA [152].

#### 3.3.4. Other Farnesoid X Receptor Agonists

Despite the therapeutic potential of OCA, severe hepatotoxicity recorded in patients with advanced liver disease or drug-related side effects, such as pruritus, are factors limiting the therapeutic use of OCA. Some of these side effects can be caused by the bile acid-like structure and behavior of the OCA molecule. Namely, enterohepatic circulation of OCA and its metabolites and significant G protein-coupled bile acid receptor 1 (TGR5) agonistic properties are challenging pharmacokinetics [153]. Thus, new non-bile acid FXR agonists are in various stages of preclinical and clinical development [154]. EDP-305, a novel non-bile acid FXR agonist, has minimal activity against TGR5. A recent experimental study reported the therapeutic efficacy of EDP-305 in direct comparison with OCA in mouse models of liver disease [155]. Phase 2 clinical trials assessing the efficacy and safety of non-bile acid-type FXR agonists, including EDP-305, tropifexor (LJN452), and cilofexor (GS-9674), in PBC patients have recently been completed; however, the results have not yet been published.

#### 3.3.5. Baricitinib

Baricitinib, a novel small molecule approved in 2018 for the treatment of moderate to severe rheumatoid arthritis, is a Janus kinase (JAK) 1 and 2 inhibitor [156]. JAK, an intracellular enzyme, responds to cytokine and growth factor receptor stimulation to affect downstream hematopoiesis and immune cell function. JAK activates signal transducers and activators of transcription (STAT) through phosphorylation, and the JAK-STAT pathway is responsible for key immune signals. A recent whole-genome linkage study suggested that JAK-STAT pathway proteins have a potential role in developing PBC [157]. A randomized, double-blind placebo-controlled trial was initiated to evaluate the efficacy and safety of baricitinib, but the study was terminated early because of low enrollment. This proof-of-concept study demonstrated that in PBC patients with a suboptimal response to UDCA, baricitinib showed a 30% decrease in ALP and improvement in inflammation and liver fibrosis markers [158].

#### 3.3.6. S-adenosyl-L-methionine (SAMe)

S-adenosyl-L-methionine (SAMe), an endogenous molecule with hepatoprotective properties linked to redox regulation and methylation, is synthesized from methionine and adenosine triphosphate (ATP). SAMe not only maintains mitochondrial function by raising glutathione levels, but also reduces fibrosis through inhibiting collagen secretion in activated HSCs [159,160,161]. In various chronic liver diseases, the SAMe biosynthesis can be decreased [162]. A prospective, open-label pilot study to evaluate the effect of SAMe and UDCA reported a significant improvement in liver biochemistry, such as ALP, GGT, total cholesterol, fatigue, and pruritus, in addition to noncirrhotic patients with PBC [163]. Through analyzing serum samples of patients treated with SAMe and an in vitro study, Kilanczyk et al., suggested that SAMe may inhibit autoimmune events in PBC patients via its antioxidant and S-glutathionylation properties [164].

#### 3.3.7. Probiotics

The use of *L. rhamnosus* GG for hepatitis, cholestasis, and fibrosis, following common bile duct ligation in mice, improved biochemical and histological indications. This can probably be explained by increased FXR activity by probiotics in the intestine. Activated FXR enhances the formation of FGF15, which reduces the production of bile acids in the liver through negative feedback. The intake of this probiotic resulted in increased activity of FXR in the intestine and increased levels of FGF15 in the blood. The use of a potent FXR antagonist blocks the positive effect of probiotics. In tissue cultures, the culture supernatant containing probiotics increases the activity of FXR and supports the relationship between probiotics and FXR activity [165].

Additionally, *L. rhamnosus* GG increases the content of Firmicutes and Actinobacteria in the gut microbiota, which converts primary bile acids into secondary bile acids, which are poorly absorbed and are consequently removed via excretion. Until now, clinical trials of probiotics in PBC have been very sparse. Considering the very encouraging results of the experimental study, further studies on this topic would potentially be very interesting.

#### 3.3.8. Mesenchymal Stem Cells

Liver transplantation (LT) is still the most essential treatment for patients with advanced, end-stage PBC. Mesenchymal stem cell (MSC) transplantation has been proposed as an effective alternative therapy for PBC patients. MSCs are fibroblast-like, multipotent cells, which can be present in almost all postnatal organs and tissues, including the liver [166]. Few studies using umbilical cord (UC) and bone marrow (BM) MSCs have been reported [167,168]. The clinical study conducted by Wang et al., demonstrated that after BM-MSC transplantation, patients’ quality of life was improved, and ALT, aspartate aminotransferase (AST), GGT and IgM significantly decreased. Additionally, histological deterioration, such as fibrosis, was not observed in BM-MSC-treated patients [168]. In addition, the beneficial effects of BM-MSCs included an increase in Tregs, a decrease in inflammatory cytotoxic CD8+ T cells, and elevated IL-10 and anti-inflammatory cytokines. Although MSC-derived immunomodulation can play a crucial role in attenuating PBC, further studies are necessary to decide the optimal frequency of MSC infusion and assess the safety of MSC-based therapy in long-term follow-ups.

#### 3.3.9. Anti-Fractalking Antibody E6011

Fractalkine (CX3CL1) is a chemokine with both chemoattractant and cell-adhesive functions, which interacts with its receptor CX3CR1 in the chemoattraction and recruitment of intraepithelial lymphocytes. Previous studies have reported that CX3CL1 in PBC could be important in the development and maintenance of portal lymphocyte infiltration in PBC [169]. Injured bile ducts resulting from PBC lead to the upregulation of CX3CL1 expression in BECs, followed by the chemoattraction of CX3CR1-expressing mononuclear cells, including CD4 (+) and CD8 (+) T cells, their adhesion to BECs, and the accumulation of biliary intraepithelial lymphocytes. E6011, a novel humanized anti-fractalkine monoclonal antibody, was developed to treat various inflammatory diseases, including Crohn’s disease, rheumatoid arthritis, and PBC [170]. Unfortunately, a clinical trial (NCT03092765) to assess the efficacy and safety of E6011 was terminated early. Although the reason for early termination is not a safety issue, it is unclear whether a new clinical trial using E6011 will be initiated.

The main results relating to the novel therapeutics mentioned above are summarized in Table 2.

## 4. Primary Sclerosing Cholangitis

PSC is a chronic hepatobiliary disease affecting the intra- and extrahepatic bile duct by multifocal fibrotic bile duct stricture, dilatation, and cholestasis. Gradual injury of the biliary tract of the liver advances to liver fibrosis, cirrhosis, and finally, end-stage liver disease [171]. The symptoms fluctuate across a broad spectrum, from asymptomatic, general symptoms (pruritus, fatigue), cholangitis, and inflammatory bowel disease (IBD), to hepatobiliary malignancies [171,172].

However, the treatment of patients with PSC is difficult, given the limited supporting data for management. Current treatment relies on individual symptom control according to the patient’s condition. The etiology affecting the intrahepatic and extrahepatic bile ducts is still unclear, even from various theories of pathogenesis and research [173]. The understanding of the pathogenesis of PSC is the first step to the development of effective therapies. New challenges for clarifying the pathogenesis and developing new target drugs are still under investigation.

### 4.1. Mechanism of Chronic Bile Duct Injury in the Bile Duct in PSC

The pathological lesion of PSC is “onion skin” scars, which appear as obliterated concentric periductal fibrosis in the lining of the bile duct cells leading to biliary stricture. How bile duct cells and immune cells (mostly T cells, neutrophils and macrophages) work together with hepatic stellate cells and portal myofibroblasts in this fibrillary production is important but unclear [174]; however, a major theory of pathogenesis is the exposure of genetically sensitive patients to environmental triggers causing bile duct injury [174,175].

#### 4.1.1. Genetics of PSC

For the predisposing factor for PSC, genetic factors contribute approximately 10% and may be explained by sibling patients who have an enhanced risk of developing PSC [176,177]. According to GWASs, the genetic architecture of PSC shares features with both autoimmune diseases and IBD [176,178]. A total of 34–60% of patients with PSC have concurrent IBD in several Western and Asian populations [179,180,181]. In addition, in up to 25% of cases, patients with PSC may have other autoimmune diseases [181]; however, PSC predisposing genes show little evidence of an association with IBD and overlap with other autoimmune diseases, such as type 1 diabetes, celiac disease, rheumatoid arthritis, sarcoidosis, multiple sclerosis, and psoriasis [175,182]. This suggests that the genetic susceptibility to PSC extends into autoimmune pathophysiology beyond that represented by IBD.

Genes in the HLA class II region encode molecules that present extracellular sources antigen to CD8+ and CD4+ T lymphocytes, supporting the adaptive immune response in disease pathogenesis of PSC as an autoimmune disease. Polymorphisms in these genes are associated with most autoimmune diseases because they contribute to the specificity of immune responses [183,184,185].

More than 20 risk genes expressed by HLA haplotypes affect the development or decrease the risk of PSC.

#### 4.1.2. Bile Acid Toxicity to Cholangiocytes and Hepatocytes

Cholangiocytes are exposed to hydrophobic bile acid in a physiological environment without cell toxicity [186]; however, hydrophobic bile acid-induced cell injury in various cell types, including hepatocytes, even at low concentrations [187]. The toxic effects of bile acids are explained by cholestasis, changes in the bile composition of disease progression in the bile ducts and colon, or impaired protective mechanisms. Bile acid is abundant in the gut, and it undergoes a bacteria-mediated transformation into bioactive molecules. Its metabolites control the host immune response by modulating the balance of Th17 and regulatory T cells (Tregs) [188]. In an observational study, conjugated primary bile acids and their derivatives were increased in patients with PSC compared with healthy controls; however, secondary bile acid did not differ between the groups [189].

The genetic variation in TGR5, promoting Cl^−^ and HCO_3_^−^ secretion, induced downregulation of the TGR5 protein in cholangiocytes of PSC patients [190]. The other gene variation stabilizing the apical cholangiocyte membrane also induced the impairment of the “biliary bicarbonate umbrella” in PSC [176]. The protective mechanism of hepatocytes against harmful bile acid accumulation is explained by FGF19, which is a negative feedback regulator of bile synthesis produced in the ileum after FXR activation by bile acids [191]. Abnormal hepatic FGF19 expression was observed in the livers of PSC patients but not in healthy controls. It induced the pathologic accumulation of bile acids in the livers of PSC patients [191,192]. Defects in homeostasis for the regulation and control of bile acid leads to the chronic progression of fibrosing cholangiopathies and hepatopathy.

#### 4.1.3. Fibrosis Development Related to Cholangiocyte and Hepatic Stellate Cell/Portal Myofibroblast

Cholangiocytes display an activated phenotype in the PSC by recognizing cytokines, hormones, and bile acids expressed by environmental damage, autoantigens, or the gut microbiome. Activated cholangiocytes are correlated with activated biliary tree stem cells that induce biliary fibrosis and progression of the bile duct [193,194]. TLR and nucleotide oligomerization domain-like receptors aid in detecting pathogens and activating bile duct cells, resulting in the secretion of proinflammatory cytokines [195]. IL-2 is a key factor in the regulation and programming of the immune system in PSC [196]. TNF-α, TGFβ1, IL-1β, and IL-6, along with CD8+ and CD4+ T cells, cause myofibroblast activation and fibrosis. These induced the peribiliary fibrosis development and subsequent cirrhosis through interactions with HSCs [197,198].

Chronic injury can lead to cholangiocyte senescence and differentiation of matrix-depositing HSCs from myofibroblasts and portal fibroblasts, resulting in tissue scarring and bile duct strictures [199,200]. During chronic senescence, the surrounding tissues are susceptible to senescence associated secretory phenotype (SASP) related damage, resulting in persistent inflammatory and fibrosis responses. Moreover, destructive SASP not only maintains the inflammatory response, but can also activate the senescent phenotype in surrounding non-senescent cells [201].

#### 4.1.4. Gut-Liver Immunity of PSC

The PSC is considered a part of the hepatobiliary manifestation of IBD, and gut-derived adaptive and innate immune responses contribute to chronic and progressive biliary inflammation. The liver biopsy with PSC showed predominant T-cell infiltration with portal inflammation. In addition, other neutrophils and macrophages have also been observed to release TGFβ chronically and contribute to chronic inflammation, fibrosis, and cirrhosis [202]. In PSCs, hepatic inflammation by nutrition or bacterial inflow through the portal tract results in aberrant hepatic expression of the mucosal vascular address in cell adhesion molecule 1 (MADCAM1) and the C-C motif chemokine ligand 25 (CCL25), which results in the recruitment of mucosal T cells to the liver [175]. Other studies of recruited T cells in the portal area reported results regarding gut and liver relationships in PSC patients with or without IBD. The normal colon expresses the endothelial adhesion molecule MADCAM1 and the chemokine CCL25, which recruit mucosal lymphocytes with receptors for MADCAM1 and CCL25 (α4β7 integrin and C-C chemokine receptor 9 (CCR9), respectively) during activation by gut dendritic cells [203,204].

The intestinal and biliary epithelia are share many properties, including the expression of tight junction proteins such as E-cadherin, pattern recognition receptors (PRRs), and the ability to release secretory IgA [205,206]. The PRR, in terms of allowing cells into gut-derived bacterial products, was expressed in Kupffer cells, sinusoidal endothelial cells, and cholangiocytes; however, in PSC patients, genetic polymorphisms reduced the threshold of PRR signaling and changed the gut microbiota, leading to liver injury [206]. Another study suggested that cellular antigen(s), which are shared by the human colon and biliary epithelium by molecular mimicry, induced immune-mediated chronic inflammation [207].

#### 4.1.5. Gut Microbial Profile in PSC

The microbiota has been considered a crucial factor for the pathogenesis of PSC in recent studies. The analysis of the gut microbiota in PSC compared with healthy controls or IBD patients has been described using 16S rRNA sequencing technologies with mucosal or fecal sources [195]. The composition of the gut microbial community is altered, with an overall reduction in bacterial diversity and altered abundance of certain bacteria compared with PSC/PSC-IBD vs. healthy controls and IBD in fecal microbiota [208,209,210,211]. Although diverse heterogeneity was observed, some bacterial taxa were consistently altered in the feces of patients with PSC compared to healthy controls. In particular, *Veillonella* was higher in the stool of PSC patients than in healthy controls in all the studies [212,213,214]. *Veillonella* discriminated PSC from healthy controls with an area under the receiver operator characteristic curve (AUC) of 0.64 [209]. They have genes that encode amine oxidases and are producers of primary amines that can act as vascular adhesion protein-1 (VAP-1) substrates, which are critical for effector cell recruitment to the liver [212,213]. An increase in the genus *Sphingomonas* expressing amine oxidase is associated with the abnormal return of intestinal lymphocytes to the liver as the basis of the gut-liver axis [212,215].

As with members of the Proteobacteria phylum, such as *E. coli*, other microbiomes, such as *Enterococcus, Streptococcus*, and *Lactobacillus,* are frequently enriched in PSCs [214,215,216]. The unit of the *Enterococcus* genus correlated with elevated levels of ALP, a marker of disease severity [211]. *Fusobacterium* was associated with intestinal inflammation severity, whereas *Enterococcus* was associated with biliary pathology [210]. Furthermore, a similar fecal microbiota composition (AUC 0.88) was analyzed in geographically different cohorts in Germany and Norway in PSC [208]. These studies presumed the possible utility of microbial components as prognostic and/or diagnostic markers in PSC.

In addition, the microbiota is being considered as a potential biological treatment option of PSC to ameliorate cholestasis and hepatic fibrosis, although with an in murine model. The PSC mice have reduced the abundance of *Prevotella copri*. The gavage P. *copri* daily in these mice induced the improvement of cholestasis in enterohepatic circulation caused by affecting bile acid level [217].

Recently, studies on the pathophysiology of PSC extended to integrative analysis between gut microbiota, gene expression, and immunologic response. Quraishi et al. insisted on microbial alteration and differential gene expression (colonic transcriptome) in PSC/IBD and ulcerative colitis patients and implicated the dysregulation of bile acid metabolism [216]. The relationship between the specific microbiome and the colonic transcriptome impacts metabolism, such as bile acid, bile salt, and fatty acid metabolism. Genomics, microbiota, and functional analysis will be considered future challenges for single microbes in disease pathogenesis; however, the microbiome is influenced by environmental factors such as diet, drugs, physical activity, hygiene, and race [214,218]. Several studies have attempted to reduce bias by controlling for antibiotic use or classifying geographic differences, but more multicenter and systematic studies are needed to control for confounding factors [210,211].

Other studies have focused on fungal dysbiosis in feces. The fungus *Exophiala,* a fungal genus of the *Herpotrichiellaceae* family, increased PSC and increased the fungal biodiversity and altered the composition [219]. Another case reported that exophiala increased liver cirrhosis through cholestasis and dilation of intrahepatic bile ducts [220].

### 4.2. Symptomatic Treatment and Biliary Complications of PSC

PSC patients are subject to several significant events throughout a fluctuating and highly variable disease course. The absence of robust data limits the standardization of treatment recommendations.

#### 4.2.1. Pruritus

Pruritus is a concomitant manifestation of cholestatic disease. More than two-thirds of these patients experience itching during the course of PSC or PBC. Increased concentrations of bile salts, histamine, serotonin, progesterone metabolites, and endogenous opioids have been controversially discussed as potential pruritogens. The pathogenesis of cholestasis pruritus remains largely unknown [221]. Recently, a randomized, placebo-controlled FITCH trial clearly showed the beneficial effect of the PPAR agonist bezafibrate (400 mg/day) on moderate to severe cholestasis-associated pruritus in PSC and PBC [222]. As another cause, the imbalance between the µ-opioid receptors (MOR) and κ-opioid receptors (KOR) has been proposed to modulate the transmission and enhancement of pruritus signals in the central nervous system. In a randomized, double-blind study of nalfurafine hydrochloride, the KOR agonist nalfurafine hydrochloride (2.5 or 5 μg daily) was effective without significant side effects in the treatment of intractable pruritus in patients with chronic liver disease [223]; however, endogenous opioid levels and MOR/KOR ligands did not differ between the levels of pruritus and nonpruritic patients with chronic liver disease and did not correlate with itch intensity [224]. The previously recommended first-line medical treatment is cholestyramine (4–16 g/day). This anion exchange resin has been reported to alleviate pruritus in several small uncontrolled case series [221]. Rifampicin, naltrexone, sertraline, and KOR agonists/MOR antagonists may be considered in the following steps; however, only a few well-designed, randomized, placebo-controlled trials and several cohort studies have evaluated these medications. Further studies to understand the correlation between pruritogen, medication and symptoms are needed in the future.

#### 4.2.2. Bacterial Cholangitis and Dominant Stricture

The risk of bacterial cholangitis is increased after endoscopic or surgical manipulations (including liver biopsy) in patients with PSC, but cholangitis can also occur spontaneously. Bile cultures from patients with PSC show a broad spectrum of bacteria in patients with and without prior biliary intervention. Although cholangitis occurs frequently, symptoms can be atypical [222]. Empirical antibiotics are typically effective, and prophylactic antibiotics should be administered before and after biliary interventions. In more severe cases, hospitalization is necessary for intravenous treatment, including broad-spectrum antibiotics [225].

In a meta-analysis study, treatment with antibiotics (metronidazole, vancomycin, rifaximin and minocycline) for PSC patients was also associated with a significant reduction in ALP and total serum bilirubin levels regardless of cholangitis [213]. Long-term antibiotic treatment (vancomycin) has been focused upon as a management method for controlling cholangitis and modulating the intestinal microflora caused by the bacterial dysbiosis of PSCs [226]. To judge the effectiveness of antibiotic treatment beyond the treatment of cholangitis, a longer treatment duration and follow-up with a powered placebo-controlled studies are needed. Furthermore, an integrative analysis should be applied to properly compare the genetic profile, microbiome, immune system, and antibiotic therapy.

#### 4.2.3. Dominant Stricture

Dominant stenosis (extrahepatic bile duct less than 1.5 mm or narrowing of less than 1 mm in the area within 2 cm of the main biliary junction) results in advanced focal stenosis in 40–58% of patients with PSC [227]. Patients with dominant stenosis may be asymptomatic and present with various clinical manifestations or with worsening liver function tests, abdominal pain, and/or cholangitis [228]. Dominant stenosis may also exacerbate symptoms of cholestasis, including worsening pruritis or developing cholangitis; therefore, endoscopic treatment with balloon dilatation or stent placement would be beneficial in dominant stenosis, however, a single retrospective observational study reported that 45% of patients with biliary stenosis showed similar changes in biochemistry for 1 year after diagnosis, with or without dominant stenosis [229]. Therefore, it has been recommended that endoscopic treatment of dominant stenosis should be performed only in the presence of clinical or biochemical abnormalities [230]; however, a recent large-scale retrospective study reported that regular endoscopic retrograde cholangiopancreatography (ERCP) with endoscopic balloon dilatation has significant benefits in PSC patients with dominant stenosis, affecting asymptomatic survival and the incidence of recurrent cholangitis [231]. In the endoscopic treatment of this dominant stenosis, balloon expansion is preferred over stent insertion because the incidence of related side effects (pancreatitis, cholangitis, bacteremia) is low [232].

#### 4.2.4. Cholangiocarcinoma

The prevalence of cholangiocarcinoma (CCA) ranges from 7 to 15% of PSC patients over the patient’s lifetime, with half of the cases being diagnosed in the first year after diagnosis of PSC, probably due to the development of CCA-related symptoms [171]. The pathogenesis of CCA in PSC is inflammation-driven carcinogenesis concomitant with various genetic and epigenetic abnormalities, but the pathogenesis of CCA in PSC is still controversial [233]. CCA may remain asymptomatic for a long time, but when symptoms appear, and CCA is found, an advanced tumor stage is usually present. Unfortunately, most cases are unresectable, and no effective medications are available when CCA is diagnosed; therefore, early recognition of CCA in PSC remains a major challenge. The surveillance and management of PSC patients at an increased risk of developing CCA are important. Several factors increase the risk of CCA in PSC patients, including old age, abnormalities related to the large duct, male sex, comorbidity of ulcerative colitis, and infections with hepatitis B or hepatitis C. Magnetic resonance cholangiopancreatography (MRCP) combined with serum CA 19–9 should be used for screening and surveillance. In contrast, ERCP-based brush cytology, biopsy, and fluorescence in situ hybridization (FISH) should be used for diagnosis [233].

#### 4.2.5. Gallbladder Carcinoma

Gallbladder disease, including gallstones or cholecystitis, is relatively common in 25% of PSC patients. A gallbladder mass lesion is found in 6–15% of PSC patients, and over half of those had adenocarcinoma after cholecystectomy [234,235]. Surveillance with regular ultrasonography of the gallbladder may be of value in these patients. Adenocarcinoma can also be found in polyps smaller than 5 mm; therefore, cholecystectomy should be considered regardless of the size of the polyp in PSC patients [236].

#### 4.2.6. Liver Cirrhosis and Hepatocellular Carcinoma, and Liver Transplantation

Histological characteristics of PSC with an “onion-skin” pattern mimicking concentric periductal fibrosis with lymphocyte infiltration and portal edema progressed to portal and bridging fibrosis. Finally, liver cirrhosis with regenerative nodules and extensive fibrosis occurred [199,200]. LT is the only curative treatment and lifesaving intervention for PSC. Indications for LT are similar to other liver diseases transplanted, according to the eligible model for end-stage liver disease (MELD) scores in cirrhotic patients. Specific indications for LT in PSC patients include recurrent uncontrollable cholangitis, decompensated secondary biliary cirrhosis, and intractable pruritus [175]; however, a high rate of recurrent PSC (more than 20%), less than 5 years after LT was observed, negatively impacted patient survival [237]. Therefore, it is important to distinguish between recurrent PSC and post-transplant biliary strictures, which can occur even more often (36%) for various reasons (ischemia, infection, treatment induction).

#### 4.2.7. Inflammatory Bowel Disease and Colorectal Cancer

In patients with PSC, IBD was observed in 60% of all patients. Among these PSC-IBD patients, colonic involvement is most often identified as ulcerative colitis. Patients with PSC-IBD have an increased risk of colorectal cancer and colorectal dysplasia, increased hepatic–pancreatic biliary cancer and mortality, and an earlier onset than patients with IBD alone [238]; therefore, PSC and PSC-IBD patients under regular surveillance have better outcomes. In patients with PSC with IBD, annual colonoscopy, including chromoendoscopy and histological sampling, is recommended regardless of the presence of symptoms of colitis [230]. In PSC patients with IBD, colonic dysplasia should be endoscopically resected, similar to management in other IBD patients. Proctocolectomy should be considered if high-grade dysplasia is discovered [230].

### 4.3. Combination Treatment and Predicting Therapies for PSC

#### 4.3.1. UDCA

UDCA is considered the first-line therapy with an excellent safety profile when administered at moderate doses in the treatment of PBC (13–15 mg/kg/day), which leads to improved LT-free survival in all patients regardless of the disease stage and biochemical response [108,239]; however, its effectiveness as monotherapy is insufficient to prevent PSC progression in most patients. Nevertheless, discontinuation of UDCA has been shown to cause worsening of liver biochemistry and symptoms [240]. This justified the maintenance of UDCA treatment. To overcome the therapeutic limitations of UDCA, a study is also underway to evaluate whether there is improvement in biliary lesions and the safety evaluation of intra-arterial injection of stem cells using UDCA together [NCT03516006].

#### 4.3.2. FXR Agonist

A poor prognosis is expected if there is no response to UDCA treatment; therefore, continuing research for drugs with a new mechanism of action is necessary. OCA acts as an FXR agonist and affects bile acid synthesis, inflammation, and liver fibrosis [190]. It has been approved as a combination therapy with UDCA when there is no response to UDCA treatment due to evidence of the improvement of biochemical profiles in a long-term phase III randomized study [108,123]. In a phase II study of single therapy OCA, there was a reduced serum ALP level in patients with PSC [241]; however, OCA-induced dose-dependent pruritus was a side effect in this study. Cilofexor, a nonsteroidal FXR agonist, also improved cholestasis and fibrosis biochemical markers in a phase II trial. A phase III trial will evaluate the reduction in the risk of fibrosis progression among noncirrhotic adults with PSC. [NCT0389012] FXR agonists induce endogenous FGF19 synthesis and affect the proliferation of hepatobiliary malignancies [242]. In this respect, long-term use of FXR agonists in PSC should require the evaluation of concomitant hepatobiliary malignancies.

#### 4.3.3. PPAR Agonist

Bezafibrate, an intranuclear receptor, acts as a ligand of PPARα. PPARα promotes the multidrug resistance 3 (MDR3) gene expression and increases P-glycoprotein levels in bile duct canaliculi, which, in turn, causes buffer damage to the bile duct by forming micelles with hydrophobic toxic bile acids [243]. Bezafibrate, which has antioxidative and anti-inflammatory effects, may reduce the damage to the bile duct epithelium seen in cholestatic diseases by inhibiting TNF-α and inducing superoxide dismutase expression [244]. In a small, randomized study, bezafibrate significantly improved the biochemistry profile in PSC patients and showed 64% efficacy. A phase III randomized trial on the effect of the biochemical profile and LT-free survival of UCDA, and the bezafibrate combination in PSC with persistent cholestasis, despite UDCA therapy, is ongoing [NCT04309773].

#### 4.3.4. 3-Hydroxy-3-methylglutaryl Coenzyme A (HMG-CoA) Reductase Inhibitor

Statins acting by inhibiting HMG-CoA reductase reduce lipid levels, leading to the inhibition of cholesterol synthesis and lowering cholesterol levels in serum and bile [245]. The effect on PSC would be decreased cholesterol levels in serum and bile and improved inflammation, bile stone formation, and ameliorated cholestasis. In a recent population-based cohort of PSC patients with IBD, statins reduced the risk for death or liver transplantation by 50%. In this study, treatment with UDCA was not associated with reduced mortality [241]. A phase III randomized trial has been ongoing for 5 years to evaluate the long-term survival and efficacy of simvastatin in improving the biochemistry profile of PSC patients [NCT04133792].

#### 4.3.5. Oral Vancomycin

Oral vancomycin, an antibiotic that is poorly absorbed into the body from the bowel and concentrates in the intestine, is also considered a treatment option for PSC. The dysbiosis of the gut microbiome affects the gut immune system, attracting white cells, which damage the bile ducts, increases the delivery of toxins to the liver through a leaky bowel wall, and directly causes damage through toxic bile acids [195,246]. From this point of view, oral vancomycin greatly modified intestinal bacteria, and in particular, caused a decrease in the number of intestinal bacteria of the *Bacteroides* and *Prevotella* species. Recently, one randomized controlled trial showed that oral vancomycin was well tolerated and was associated with improvements in liver chemistry [247]. A phase III study will evaluate the efficacy of oral vancomycin in treating biochemistry and fibrosis in PSC [NCT03710122].

In addition to these studies, various novel therapies are being conducted to inhibit the progression of PSC through chemokine receptor antagonists, FGF19 analogs or fecal microbiota transplantation (Table 3).

**Figure 1:**
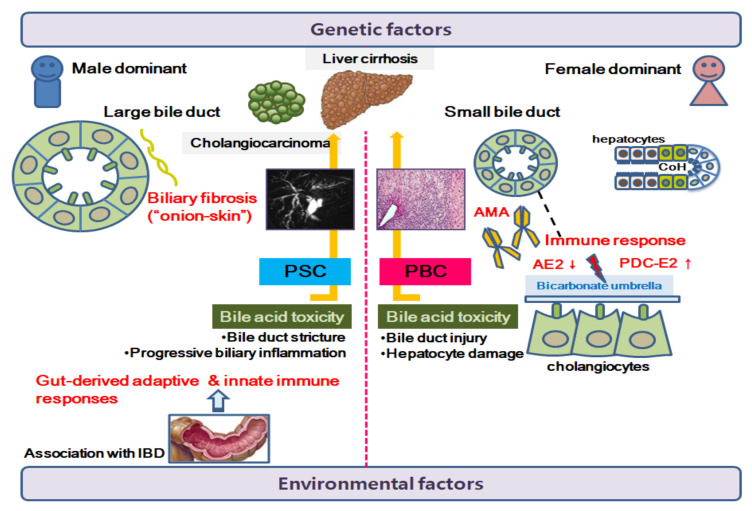
The comparison between PBC and PSC. This is a figure highlighting the differences between two diseases. The picture is adapted from Gidwaney et al. [254] and Libre Pathology (https://librepathology.org, accessed on 28 April 2022). CoH: canals of Hering, PSC: primary sclerosing cholangitis, PBC: primary biliary cholangitis, IBD: inflammatory bowel disease, AMA: anti-mitochondrial antibody, AE2: anion exchanger 2, PDC-E2: pyruvate dehydrogenase complex, E2.

Hitherto, we have covered the pathophysiological mechanisms, clinical features, and treatment of PBC and PSC. Figure 1 shows the differences between PBC and PSC.

## 5. Conclusions

Despite extensive research to understand the molecular pathogenic features of PBC and PSC, there are still significant gaps between knowledge and its application to clinical practice. As medical treatments, UDCA and OCA have been approved for PBC; however, patients who are not responsive to these treatments remain a challenge. Moreover, medical therapy for PSC remains limited, with no effective or approved pharmacologic treatment identified. Fortunately, various therapeutics targeting pathogenic molecules in these cholestatic liver diseases are under investigation. Combination therapy composed of anti-biliary, anti-inflammatory, antioxidative, or anti-fibrotic treatment may be a viable solution. It is necessary to identify patients at high risk for disease progression or malignancy. Through continual research, we will improve treatment options for these complex and perplexing chronic progressive hepatobiliary diseases.

## Figures and Tables

**Table 1:** Response criteria of UDCA therapy in patients with PBC.

Criteria	Definition of Response (Time to Evaluation, Months)	Ref.
Barcelona	>40% decrease or normalization of ALP (12)	[109]
Mayo	ALP < 2 × ULN (6)	[110]
Paris I	ALP ≤ 3 × ULN and AST ≤ 2 × ULN and normalization of bilirubin (12)	[111]
Rotterdam	Normalization of bilirubin and/or albumin (12)	[112]
Ehime	≥70% decrease or normalization of GGT (6)	[113]
Toronto	ALP ≤ 1.67 × ULN (24)	[114]
Paris II	ALP and AST ≤ 1.5 × ULN and normalization of bilirubin (12)	[115]

ALP: alkaline phosphatase, ULN: upper limit of normal, GGT: γ-glutamyl transferase, AST: aspartate aminotransferase.

**Table 2:** Novel therapies currently under investigation in PBC.

Agent	Mechanism	Clinical Trial Stage	Main Results	Ref.
Bezafibrate	Panspecific PPAR agonist	Phase III (BEZURSO trial)	Complete biochemical response: 31% vs. 0%; ALP normalization: 67% vs. 2%; Improvement in liver stiffness: 15% decrease vs. 22% increase.	[94]
Elafibranor	PPARα and PPARδ agonist	Phase II (NCT03124108)	Significant decrease in ALP: 41–48% vs. 3% increase; ALP < 1.67 × ULN, ALP decrease > 15%, total bilirubin < ULN: 67–79% vs. 6.7%.	[140]
Saroglitazar	PPARα and PPARγ agonist	Phase II (EPICS)	Mean percentage reduction in ALP: 48.9–50.6% vs. 3.3%; ALP < 1.67 × ULN, ALP decrease > 15%, total bilirubin < ULN: 69–71% vs. 10%.	[141]
Tropifexor	FXR agonist	Phase II (NCT02516605)	Not yet published	
Cilofexor	FXR agonist	Phase II (NCT02943447)	Not yet published	
EDP-305	FXR agonist	Phase II (NCT03394924)	Not yet published	
Baricitinib	JAK 1 and 2 inhibitor	Proof-of-concept study	Thirty percent decrease in ALP; Improvement in the itch NRS; Increase in the fatigue NRS; Improvement of inflammation and liver fibrosis marker.	[158]
S-adenosyl-L-methionine	17-beta-estradiol glucuronide-induced cholestasis reversal agent	Pilot, open-label study	A positive effect of adding SAMe to UDCA in noncirrhotic PBC patients.	[163]
Probiotics	Regulation of bile acid homeostasis	Phase II (NCT03521297)	Data yet to be collected	
Mesenchymal Stem Cells	Immunoregulation	NCT03668145	Data yet to be collected	

PPAR: Peroxisome proliferator-activated receptor, ALP: alkaline phosphatase, ULN: upper limit of normal, SAMe: S-adenosyl-L-methionine, UDCA: Ursodeoxycholic acid, PBC: primary biliary cholangitis, FXR: farnesoid X receptor.

**Table 3:** Novel therapies currently under investigation in PSC.

Agent	Mechanism	Clinical Trial Stage	Main Results	Ref.
24-norursodeoxycholic acid (norUDCA)	Side chain-shortened C23 homolog of UDCA	Phase II (NUC-3) Phase III (NCT03872921)	Reduced ALP levels by −12.3%, −17.3%, −26.0% in the 500, 1000, 1500 mg/d groups. Data yet to be collected	[248]
Berberine ursodeoxycholate (BUDCA)	Ionic salt of two active moieties, berberine and UDCA	Phase II	Not yet published	
Obeticholic acid	FXR agonist	Phase II (AESOP)	Reduced serum ALP.	[249]
Cilofexor	FXR agonist	Phase III (PRIMIS)	Data yet to be collected	
Vidofludimus calcium	FXR agonists + dihydroorotate dehydrogenase inhibitor	Phase II	Normalization of ALP occurred in 27.7%.	[250]
Benzafibrate (+UDCA)	PPAR agonist	Phase III (BEZASCLER)	Data yet to be collected	
Seladelpar	PPARδ agonist	Phase II (NCT04024813)	Not yet published	
Simvastatin	HMGCoA reductase inhibitors	Phase III (PiSCATIN)	Data yet to be collected	
Vancomycin	Antibiotics	Phase III (NCT03710122)	Data yet to be collected	
CM-101	Monoclonal Ab blocking CCL24	Phase II (SPRING)	Data yet to be collected	
Cenicriviroc	Dual antagonist of CCR2 and CCR5	Phase II (PERSEUS)	Median 18% reduction in ALP.	[251]
Timolumab	Monoclonal anti-VAP-1 antibody	Phase II (BUTEO)	Not yet published	
NGM282	FGF19 analog	Phase II	Enhanced Liver Fibrosis score and inhibited bile acid synthesis without affecting ALP levels.	[252]
Sulfasalazine	Aminosalicylates	Phase II (NCT02177136)	Data yet to be collected	
Fecal Microbiota Transplantation	Restore the microbiome	Phase II	Thirty percent experienced a ≥ 50% decrease in ALP.	[253]
Umbilical Cord Mesenchymal Stem Cells	Repair of damaged tissue and immuno-modulation	Phase II (NCT03516006)	Data yet to be collected	

UDCA: Ursodeoxycholic acid, PPAR: Peroxisome proliferator-activated receptor, ALP: alkaline phosphatase, FXR: farnesoid X receptor, HMG-CoA: β-hydroxy β-methylglutaryl-CoA, CCL: C-C motif chemokine ligand, CCR: C-C motif chemokine receptor.
